# Draft Genome Sequence of Filamentous Fungus *Phialemoniopsis curvata*, Isolated from Diesel Fuel

**DOI:** 10.1128/MRA.00400-19

**Published:** 2019-07-25

**Authors:** Vanessa A. Varaljay, Wanda J. Lyon, Audra L. Crouch, Carrie A. Drake, Jeffrey M. Hollomon, Lloyd J. Nadeau, Heather S. Nunn, Bradley S. Stevenson, Caitlin L. Bojanowski, Wendy J. Crookes-Goodson

**Affiliations:** a Soft Matter Materials Branch, Materials and Manufacturing Directorate, Air Force Research Laboratory, Wright-Patterson AFB, Ohio, USA; b UES, Inc., Dayton, Ohio, USA; c 711th Human Performance Wing, Airman Systems Directorate, Air Force Research Laboratory, Wright-Patterson AFB, Ohio, USA; d Department of Microbiology and Plant Biology, University of Oklahoma, Norman, Oklahoma, USA; University of California, Riverside

## Abstract

Phialemoniopsis curvata D216 is a filamentous fungus isolated from contaminated diesel fuel. The genome size is 40.3 Mbp with a G+C content of 54.81%. Its genome encodes enzymes and pathways likely involved in the degradation of and survival in fuel, including lipases, fatty acid transporters, and beta oxidation.

## ANNOUNCEMENT

Here, we describe the draft genome sequence for the filamentous fungus Phialemoniopsis curvata strain D216, isolated from a biofouled diesel fuel storage tank. Filamentous fungi, such as *Byssochlamys* spp. and *Fusarium* spp., are known biocontaminants of biodiesel and jet fuel (1–3). *Phialemoniopsis* is a newly described genus of the *Sordariomycetes* previously found only in soil environments and clinical samples (4) and for which there are currently no sequenced genomes. *P. curvata* strain D216 grows on B20 biodiesel as its sole carbon source and forms a pellicle-like biofilm at the water-fuel interface. The sequenced genome of *P. curvata* strain D216 provides the first genome for an isolate of this genus and will further our understanding of how this organism grows in fuel systems.

D216 was originally isolated from a contaminated diesel fuel tank and grown on potato dextrose agar (PDA). For whole-genome sequencing, D216 was inoculated from filaments from PDA plates and grown in tryptic soy broth for 2 days with shaking (200 rpm) at 27°C. The biomass was pelleted, flash frozen in liquid nitrogen, and stored at –80°C. Genomic DNA was extracted using the Qiagen Genomic-tip system following a user-developed protocol (see https://www.qiagen.com/us/resources/resourcedetail?id=cb2ac658-8d66-43f0-968e-7bb0ea2c402a&lang=en). Extracted genomic DNA was sequenced as 4 individually prepared libraries on an Illumina MiSeq run using 300-bp paired-end (PE) read sequencing.

Sequencing resulted in 19 million raw PE reads from 4 libraries, which were trimmed and quality filtered with Trimmomatic v0.36 (5). The 18 million (36 million total) trimmed, quality-filtered PE reads (226 ± 19 bp) were assembled using SPAdes v3.12.0 (6), resulting in ∼100× coverage and 263 contigs with a G+C content of 54.81%, an *N*_50_ value of 377,547 bp, an *L*_50_ value of 36, and a genome size of 40.3 Mb, as calculated using QUAST v4.6.3 (7). The D216 genome completeness was estimated to be 98.1% using Benchmarking Universal Single-Copy Orthologs (BUSCO) v3 (8) and the *Sordariomycetes* database as a reference. The assembled genome was masked using RepeatMasker (9) with Repbase (10) and whole-genome *de novo* gene prediction performed with AUGUSTUS v3.3.1 (11) using Fusarium graminearum as the model organism. This prediction resulted in 12,491 putative genes. The genome was annotated with Blast2GO v5 (12) using BLASTP against Uniprot (10^−5^ E value cutoff) and using InterProScan against the InterPro collection of databases. A comparison of the 28S rRNA (D1 to D2 region), internal transcribed spacer (ITS) region, 18S rRNA, actin, and β tubulin genes showed that the D216 sequences were 100% identical to those available from *Phialemoniopsis curvata* strain CBS 490.82 (4).

Based on BLASTP and KEGG analyses using GhostKOALA (13), we identified putative enzymes and pathways likely involved in fuel degradation and survival. We identified putative enzymes which could be involved in the degradation of fuel, including Yarrowia lipolytica Lip2p lipase homologs (14), cutinases, and acetylxylan esterases. The presence of putative homologs for fatty acid uptake transporters, Fat1p, Faa1p, and Faa2p (15), and the beta oxidation pathway for fatty acid metabolism further suggests that D216 is capable of survival in fuel. This sequenced genome should provide critical insights into the metabolism and physiology leading to biofouling and degradation of diesel fuel by this filamentous fungus.

### Data availability.

The whole-genome shotgun sequencing project has been deposited at GenBank under accession number SKBQ00000000. The raw data reads have been deposited in the Sequence Read Archive (SRA) database under the accession numbers SRX5630381, SRX5630382, SRX5630383, and SRX5630384.
